# Dynamics of Competition between Subnetworks of Spiking Neuronal Networks in the Balanced State

**DOI:** 10.1371/journal.pone.0138947

**Published:** 2015-09-25

**Authors:** Fereshteh Lagzi, Stefan Rotter

**Affiliations:** Bernstein Center Freiburg and Faculty of Biology, Freiburg, Germany; Georgia State University, UNITED STATES

## Abstract

We explore and analyze the nonlinear switching dynamics of neuronal networks with non-homogeneous connectivity. The general significance of such transient dynamics for brain function is unclear; however, for instance decision-making processes in perception and cognition have been implicated with it. The network under study here is comprised of three subnetworks of either excitatory or inhibitory leaky integrate-and-fire neurons, of which two are of the same type. The synaptic weights are arranged to establish and maintain a balance between excitation and inhibition in case of a constant external drive. Each subnetwork is randomly connected, where all neurons belonging to a particular population have the same in-degree and the same out-degree. Neurons in different subnetworks are also randomly connected with the same probability; however, depending on the type of the pre-synaptic neuron, the synaptic weight is scaled by a factor. We observed that for a certain range of the “within” versus “between” connection weights (bifurcation parameter), the network activation spontaneously switches between the two sub-networks of the same type. This kind of dynamics has been termed “winnerless competition”, which also has a random component here. In our model, this phenomenon is well described by a set of coupled stochastic differential equations of Lotka-Volterra type that imply a competition between the subnetworks. The associated mean-field model shows the same dynamical behavior as observed in simulations of large networks comprising thousands of spiking neurons. The deterministic phase portrait is characterized by two attractors and a saddle node, its stochastic component is essentially given by the multiplicative inherent noise of the system. We find that the dwell time distribution of the active states is exponential, indicating that the noise drives the system randomly from one attractor to the other. A similar model for a larger number of populations might suggest a general approach to study the dynamics of interacting populations of spiking networks.

## Introduction

Animal behavior emerges as a consequence of neuronal interactions and their plastic changes during learning. Self-organization through plasticity is what makes neuronal networks capable of performing computations that serve their biological function. It has long been hypothesized that groups of neurons interact to achieve higher cognitive functions. It is therefore important to analyze how groups of neurons dynamically cooperate with each other, and what are the emergent properties of such interactions. In the crudest simplification, there are just two types of neurons which mainly establish either excitatory or inhibitory synapses with other neurons (Dale’s principle [1]). Moreover, it is believed that in the mammalian neocortex there is a non-random sparse connectivity between neurons [2] and there are clustered structures with stronger synapses and increased connection probability [3], also known as cell assemblies. Moreover, it has been demonstrated that, in the mammalian sensory cortices, Hebbian learning can increase or decrease the strength of connections between neurons with similar stimulus preferences [4–7].

Theoretical studies have demonstrated that one of the possible by-products of clustered connectivity is the emergence of bi/multi-stability (and as a possible consequence, switching dynamics) between interacting populations [8–12]. There are many examples of bistable phenomena in biological neural networks, such as decision making, binocular rivalry, as well as spontaneous cortical state switchings (up and down states), particularly noticeable under certain types of anesthesia. In the following, we point out some examples wherein an attractor network with bistable property can explain the collective dynamics.

The emergence of bistability in an attractor network may serve as a possible explanation for some of the phenomena involving switching behavior described in experiments, particularly for processes involved in cognitive functions such as decision-making [10, 11]. Neuronal population recordings obtained from decision-associated cortical areas (most prominently from the lateral intraparietal cortex (LIP) [13]) of awake macaque monkeys during the performance of perceptual discrimination tasks have revealed a high correlation between changes in single cell and population activity and the relevant aspects of the animal’s behavior, in particular response time and perceptual choice [14–16]. To account for these observations, previous studies have explored simple, binary attractor models of spiking neural networks, composed of distinct sub-populations [17, 18]. In these models, each attractor corresponds to a distinct alternative of the decision. Furthermore, it has been reported that an attractor network derived from the envisaged energy landscape could, in fact, reproduce the statistics of the competition dynamics [19].

Apart from studies on decision-making, there are other studies indicating that neuronal population interactions, and particularly competition between them correlates with many interesting behaviors. It was shown that neuronal activity in the visual cortex correlates with the perceived image during binocular rivalry [20], a phenomenon that occurs when the two eyes are exposed to different stimuli. Under this condition, there is a switching dynamics between the perceived stimuli, and the duration of the percept appears to be stochastic [21]. Reciprocal inhibition between competing neurons has been shown to be fundamental for the emergence of perceptual switching in binocular rivalry [22, 23]. As another example, in the auditory system, in the presence of ambiguous stimulation, bistable perception occurs. There are strong similarities between these sensory systems, suggesting a common principle behind bistable perception [24].

There is also research on the occurrence of elevated persistent activity, during the delay period in working memory tasks, which has been hypothesized to be based on the emergence of attractors in the underlying network [25–27]. In these studies, an external drive is used to change the network activity and, after the stimulus suppression, the trajectory is attracted to a different stable fixed point, corresponding to the elevated persistent activity commonly reported in working memory tasks [28, 29]. In balanced random networks, this kind of dynamics is induced by nonlinear interactions between groups of neurons. Experiments on localization and short term memory in behaving monkeys have demonstrated that there are well-separated states of activity within which the firing rates are stationary, and cortical activity switches between these states depending on the stimulus and the behavioral conditions [30]. These global state switches are also highly prominent during the different stages of the sleep cycle, and under the effect of certain anesthetic agents [31–33]. The stability of these different states as well as the transitions between them are achieved by the cooperative and concerted activity of many neurons, making it a collective phenomenon and potentially supporting the idea of attractor networks as the underlying cause.

Apart from switching dynamics, population interactions under specific conditions result in oscillatory dynamics that are crucial, for example, for Central Pattern Generators in lower parts of the nervous system [34], and for the emergence of gamma-band oscillations in cortex [35–37]. Given the great variety of different dynamical behaviors that can emerge from population interactions, it is interesting to come up with a general mathematical framework that is able to reproduce all these dynamics by changing parameters, and that can be matched to the population dynamics of spiking neuronal networks. One major problem in analyzing the collective behavior of spiking neural networks is the nonlinear neuronal spiking dynamics due to threshold, reset and refractory period. A well-known model of population interaction is based on the concept of an event density in renewal theory and employs sigmoid input-output functions of neural populations when appropriate coarse graining is applied [38, 39]. Threshold-linear dynamics [40] and integral equations based on renewal theory [41] are alternative methods of describing and analyzing interacting populations. Each model makes specific assumptions about neural dynamics, and is valid only for a specific range of time-scales. However, in order to be able to describe phenomena like switching dynamics or limit cycle solutions, the dynamical equations have to be nonlinear. Wilson-Cowan equations [39] are able to replicate the above-mentioned dynamics for specific parameters. However, it is not clearly understood how these parameters can be determined from a known network structure and node dynamics.

In this article, we introduce a new type of dynamical equations based on competition and cooperation between interacting subnetworks of spiking neurons. The idea behind the model comes from a well-known mathematical equation for population dynamics in ecosystems where preys and predators interact, typically one feeding on the other. The basic type of such predator-prey models were suggested by Lotka [42, 43] and Volterra [44] and therefore are known as (generalized) Lotka-Volterra equations. It was shown that they are powerful enough to represent nonlinear polynomial equations and the issue of recasting a general nonlinear system into generalized Lotka-Volterra equations were discussed [45]. In this paper, we aim at modeling the interaction between subnetworks using this mathematical framework, by deriving the equations from the solution of the stationary first-passage time problem for leaky integrate-and-fire neurons. Then, a first order temporal perturbation of the system will result in the desired dynamics. Lotka-Volterra equations are generally capable of producing limit cycle and chaotic solutions, as well as switching dynamics. They seem to be rich enough to approximate also neural population dynamics. They have been suggested as dynamical equations of single neurons [46] and were already shown to be able to represent winner take all dynamics. However, to the best of our knowledge, these equations as a collective dynamics of spiking neural networks have not been justified analytically.

Lotka-Volterra equations have previously been suggested as a mechanism to generate metastable systems with saddle nodes [47, 48] that can exhibit winnerless competition dynamics. This framework allows robust transient dynamics [49] that was hypothesized to underly sensory encoding in the olfactory system [50–52]. In the case studies considered here, which are composed of three interacting populations in a symmetric network, the Lotka-Volterra equations in a two-dimensional reduction show two stable fixed points. Therefore, the dynamics support attractor models. Analysis in higher dimensions and with more interacting populations, however, can result in more complicated nonlinear dynamics.

Attractor models are simple and intuitive models that can account for the switching dynamics between populations [10, 18, 19, 53]. It has been recently shown that they are capable of representing neural variability that is also found in electrophysiological recordings during evoked and spontaneous states [8]. However, the deterministic dynamics of such models per se cannot represent “spontaneous” transitions between states. Therefore, it is essential to assume the existence of an additional stochastic component. This random noise can either come from an external source or it may derive from the intrinsic fluctuations inside the network, typically modeled as a self-generated noise of the system. This intrinsic noise can, for example, be due to finite size effects, and it has indeed been shown that rescaling the size of the neuronal populations in spiking networks can affect the switching dynamics [19, 54].

In this article, we consider two different cases of three interacting populations of spiking networks. First, we look at a network with two excitatory subnetworks with a shared inhibitory subnetwork (EEI network). Then, we consider a network with two interacting inhibitory subnetworks that interact with a common excitatory subnetwork (EII network). We are most interested in the regime where switching dynamics between the activities of the competing populations emerges. In the Methods section, we derive Lotka-Volterra equations from a first-order temporal perturbation of the stationary solution of the leaky-integrate and fire model using a standard mean-field approach. We employ numerical simulations of a spiking network and compare its behavior to our theory. In the Results section we demonstrate that the variable corresponding to the single subnetwork can be expressed in terms of the dynamics of the other two competing subnetworks. The resulting two-dimensional system has two attractors which are separated by a saddle node. In both case studies, in fact, the nullclines of the analytical equations match with those extracted from the time series of network simulations. Moreover, it turns out that the life time distributions of single population activity states are all approximately exponential. This provides evidence for a prominent role of random fluctuations (“noise”) in perturbing the trajectories. We will also show that the inherent noise of the system is multiplicative, as was demonstrated before [55], and that the whole system is in a balanced state with a high correlation between inhibitory and excitatory population firing rates.

## Materials and Methods

We aim at characterizing the dynamics of competition between interacting excitatory and inhibitory neuronal subnetworks. Two different scenarios of three interacting subnetworks will be studied analytically and the results will be verified by numerical simulations. We will show that generalized Lotka-Volterra equations with a constant term could be inferred using standard statistical methods. These equations provide a good description for the slow dynamics (order 100 Hz in the frequency domain) of the collective firing rates of subnetworks.

### Network structure and parameters

**Fig 1:**
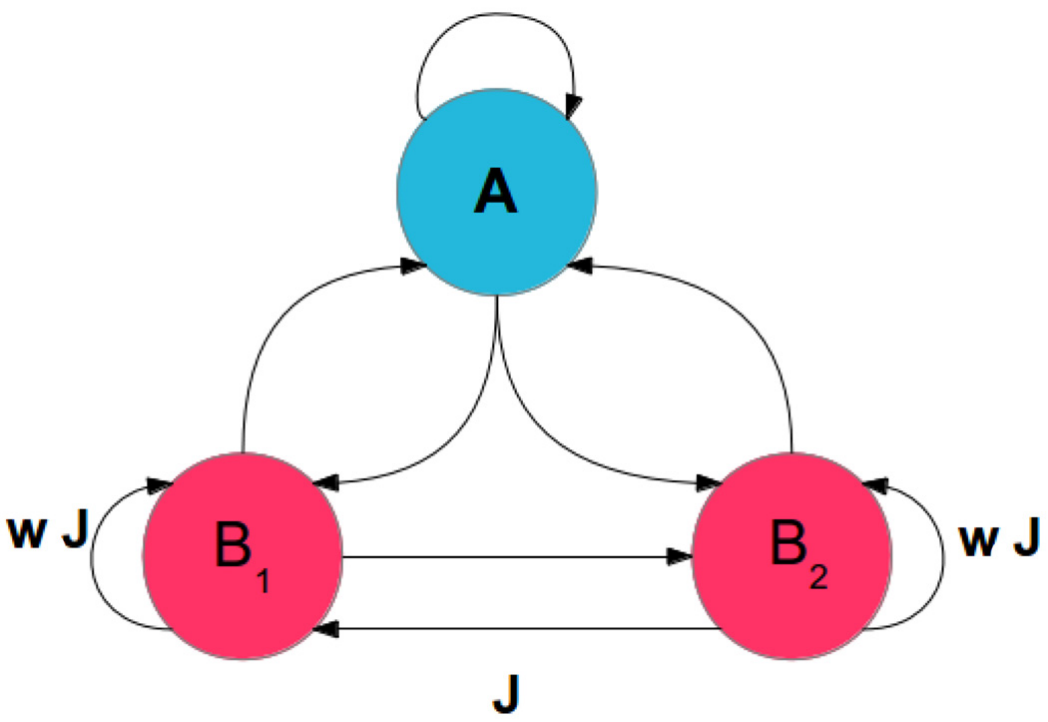
Subnetworks and connections between them to form the full network. The subnetworks are comprised of either excitatory or inhibitory neurons. Subnetworks *B*_1_ and *B*_2_ consist of neurons of the same type, either excitatory (the EEI scenario) or inhibitory (the EII scenario). The subnetwork *A* has neurons of the opposite identity. For excitatory (inhibitory) *B*_1_ and *B*_2_, we arrange *w* ≥ 1 (*w* ≤ 1).

The network consists of 4 000 excitatory neurons and 1 000 inhibitory neurons. We used leaky-integrate-and-fire neuron models with a time constant of *τ* = 20 ms. A reset potential of 10 mV and a threshold value of *θ* = 20 mV was chosen for each neuron. The dynamics of the membrane potential *v*_*i*_(*t*) of neuron *i* in the network obeys

$$\begin{array}{c} \tau {\dot{v}}_{i}\left(t\right)=-v_{i}\left(t\right)+\tau \sum_{j=1}^{N}J_{ij}S_{j}\left(t-t_{d}\right) \end{array}$$ (1)

where *S*_*j*_ is the spike train of neuron *j*, which projects to the post-synaptic neuron *i* with a delay of *t*_*d*_. Throughout the paper, *t*_*d*_ = *dt* = 0.1 ms, where *dt* is the time resolution for network simulation. Each neuron receives an external DC current of 270 pA. We decided to apply a DC current instead of a stationary Poissonian spike train as an external input to each neuron because, for our study, any external source of randomness, which could impose the observed symmetry breaking, was to be avoided. However, formally, in the dynamics of the membrane potential, we use the equivalent Poisson rate for this external input. We consider two different scenarios in which two populations with neurons of the same type (excitatory or inhibitory) are competing with each other, and the third population comprised of neurons of the different identity is coupled symmetrically to the competing populations. The schematic of the network is shown in Fig 1, where subnetworks *B*_1_ and *B*_2_ have the same type of neurons and subnetwork *A* consists of neurons of the opposite type. Throughout the paper we consider networks of fixed in-degree and fixed out-degree to avoid heterogeneities in number of connections and emergence of hubs in the network. To this end, we used a variant of a configuration model [56, 57] to derive the connectivity matrix between neurons in the entire network. For a network of three subnetworks, 9 blocks of fixed column sum and fixed row sum were generated for the adjacency matrix of connectivity. We did not allow any self-connections or multiple connections between neurons. All subsequent network simulations were conducted with NEST [58].

#### The EEI scenario

There are two subnetworks of 2 000 excitatory neurons each, with identical parameters. Each excitatory subnetwork receives local recurrent input from its own presynaptic population which comprises 10% of the whole respective population. The number of outgoing connections from each neuron to each excitatory population is also set to contact 10% of all neurons in the corresponding population. EPSP amplitude for inputs from neurons residing in the competing excitatory population is *J* = 0.1 mV. For inputs from within each population, we impose a larger amplitude *wJ* = 0.1*w* mV, where *w* is a factor between 1.0 and 3.0. This way, each excitatory subnetwork is distinct from the other one only by stronger internal connections between neurons.

In addition to the two types of excitatory inputs, each excitatory neuron receives input from 300 randomly selected inhibitory neurons from an inhibitory subnetwork of total size 1000 neurons. The IPSP amplitude is *g* = 6 times larger than the EPSP amplitude. Each neuron in the inhibitory population receives input from 30% of each excitatory population. The EPSP amplitudes are equal to *J* = 0.1 mV, identical with the other connections between excitatory populations. The local inhibitory connections inside the inhibitory subnetwork are established with a fraction of 30% of the whole population [59].

#### The EII scenario

In the second scenario we study in this paper, the network has two inhibitory populations of size 500 neurons each, that are competing. There are 4 000 excitatory neurons in one homogeneous population, each of which projects to 150 randomly selected inhibitory neurons in each of the inhibitory populations. The probability of connection between two randomly chosen neurons from the inhibitory population is 30%. Each inhibitory neuron also projects to 30% of all excitatory neuron. The IPSP to EPSP ratio is *g* = 6 and the EPSP amplitude is *J* = 0.1 mV. Neurons within each inhibitory population have IPSP amplitude equal to *wgJ*, where *w* is a factor between 0.0 and 1.0. As a consequence, inhibitory neurons within each subnetwork are less strongly coupled to each other, unlike the scenario described above, where *w* was a factor greater than or equal to 1.0.

#### Dimensionality reduction and inter-population couplings

To reduce the dimensionality of the described model, we considered the population spike train of each subnetwork as a signal that represents the activity of the population. The population spike trains of the three sub-networks were sampled in time bins of size 10 ms (too large time bins distort the temporal dynamics and, too small bins distort the statistics of the population signals), yielding estimates of the time dependent firing rates of each population. In other words, we used a time dependent histogram of the spike counts of the population activities (to which we refer as “population histogram” in the rest of the manuscript). To visualize the slow changes in the dynamics of the rates, we smoothed the time series using a Savitzky-Golay filter [60], which are derived from polynomial regression, with *n* = 10 and *m* = 4, where 2*n* + 1 and *m* are the length of the filter in discrete time and the order of the polynomial used for regression, respectively. The main advantage of this filter is that it does not distort the relative maxima and minima of the signal.

To specify the contribution of each subnetwork to the neural dynamics, for individual neurons in each population, Eq (1) can be re-written in the following form

$$\begin{array}{c} \tau {\dot{v}}_{1}\left(t\right)=-v_{1}\left(t\right)+\mu_{1}\left(t\right)+\sqrt{\tau }\sigma_{1}\left(t\right)\xi_{1}\left(t\right) \end{array}$$

$$\begin{array}{c} \tau {\dot{v}}_{2}\left(t\right)=-v_{2}\left(t\right)+\mu_{2}\left(t\right)+\sqrt{\tau }\sigma_{2}\left(t\right)\xi_{2}\left(t\right) \end{array}$$

$$\begin{array}{c} \tau {\dot{v}}_{3}\left(t\right)=-v_{3}\left(t\right)+\mu_{3}\left(t\right)+\sqrt{\tau }\sigma_{3}\left(t\right)\xi_{3}\left(t\right) \end{array}$$

where in the EEI scenario, indexes 1 and 2 refer to the two competing excitatory populations and index 3 refers to the inhibitory population. In the above equations, *μ*_*k*_ and *σ*_*k*_, *k* ∈ {1,2,3} represent the first and second moments of the input to each neuron in its corresponding population. *ξ*_*i*_ is a stochastic variable that represents the fluctuations of the input and it has the properties of a white noise with zero autocorrelation for non-zero lags. It is straightforward to verify that the input statistics can be inferred from the following equations [61]

$$\begin{array}{ccc} \mu_{1}\left(t\right) & = & \tau (N_{E}\epsilon wJr_{1}\left(t\right)+N_{E}\epsilon Jr_{2}\left(t\right)-N_{I}p\epsilon gJr_{3}\left(t\right)+Jr_{0}\left(t\right))\\ \mu_{2}\left(t\right) & = & \tau (N_{E}\epsilon Jr_{1}\left(t\right)+N_{E}\epsilon wJr_{2}\left(t\right)-N_{I}p\epsilon gJr_{3}\left(t\right)+Jr_{0}\left(t\right))\\ \mu_{3}\left(t\right) & = & \tau (N_{E}p\epsilon Jr_{1}\left(t\right)+N_{E}p\epsilon Jr_{2}\left(t\right)-N_{I}p\epsilon gJr_{3}\left(t\right)+Jr_{0}\left(t\right)) \end{array}$$ (2)

and

$$\begin{array}{c} \sigma_{1}^{2}\left(t\right)=\tau J\left(N_{E}\epsilon w^{2}Jr_{1}\left(t\right)+N_{E}\epsilon Jr_{2}\left(t\right)+N_{I}p\epsilon g^{2}Jr_{3}\left(t\right)\right) \end{array}$$

$$\begin{array}{c} \sigma_{2}^{2}\left(t\right)=\tau J\left(N_{E}\epsilon Jr_{1}\left(t\right)+N_{E}\epsilon w^{2}Jr_{2}\left(t\right)+N_{I}p\epsilon g^{2}Jr_{3}\left(t\right)\right) \end{array}$$

$$\begin{array}{c} \sigma_{3}^{2}\left(t\right)=\tau J\left(N_{E}p\epsilon Jr_{1}\left(t\right)+N_{E}p\epsilon Jr_{2}\left(t\right)+N_{I}p\epsilon g^{2}Jr_{3}\left(t\right)\right) \end{array}$$

where *r*_0_(*t*) is the equivalent external rate for the external DC current input for each neuron. (Note that we did not apply an external Poisson spike train to avoid a possible impact of the external noise on the switching dynamics. Also, note that *r*_0_ does not enter the equations for *σ*_*k*_.) *N*_*E*_ and *N*_*I*_ are the sizes of the excitatory and inhibitory populations, and *p* is a factor that will be multiplied by *ϵ* to indicate a higher probability of connections when it is needed. Throughout the paper, this parameter is fixed and equal to 3. Considering the fact that we are studying slow dynamics of the network, it is viable to assume the firing rate of each population as the sum of the firing rates of its components. It implies that $r_{i}=\frac{R_{i}}{N_{i}}$, where *R*_*i*_, *i* ∈ {1,2,3} is the population rate variable. From the above equations it is easy to verify that in Eq (2), the matrix that links the firing rates (*R*_1_, *R*_2_, *R*_3_) to the mean membrane potentials of the neurons in the corresponding subnetworks is

$$\begin{array}{c} W_{EEI}=\tau \epsilon J(\begin{array}{ccc} w & 1 & -pg\\ 1 & w & -pg\\ p & p & -pg \end{array}) \end{array}$$ (3)

Similarly, a coupling matrix could be derived for the EII network, in which *R*_1_ and *R*_2_ are population firing rates of the two competing inhibitory populations and *R*_3_ is the firing rate of the excitatory population. This matrix is of the following form

$$\begin{array}{c} W_{IIE}=\tau \epsilon J(\begin{array}{ccc} -pwg & -pg & p\\ -pg & -pwg & p\\ -pg & -pg & 1 \end{array}) \end{array}$$ (4)

In the next section, we will use these matrices in the competition-based equations for the mean-field dynamics of the network.

### Competition model of activity

A well-studied model of competition between different animal species (e.g. predators and preys) in an ecosystem is a set of coupled differential equations known as Lotka-Volterra equations [42–44]. It is known that nonlinear ODEs could have a “canonical” form resembling Lotka-Volterra equations [45]. Inspired by the rich dynamics of such systems in modeling competitive behavior, and the fact that competition is also a frequent behavioral pattern in neuronal systems, we hypothesize a variant of Generalized Lotka-Volterra equations that can capture the network dynamics. In order to justify the Lotka-Volterra (LV) equations as a model for the dynamics of neural populations, we consider a first-order perturbation of the (approximate) firing rate of a single neuron in the low firing rate regime [61]. The low-frequency dynamics of the firing rate of neuron *i* in any given population is

$$\begin{array}{c} \iota {\dot{r}}_{i}\left(t\right)=-r_{i}+\frac{\theta -\mu }{\sigma \tau \sqrt{\pi }}exp\left(-\frac{{\left(\theta -\mu \right)}^{2}}{\sigma^{2}}\right) \end{array}$$ (5)

where $\mu =\sum_{i=0}^{3}k_{i}r_{i}$ and $\sigma^{2}=\sum_{i=1}^{3}|J_{i}k_{i}|r_{i}$ are the mean and the variance of the input to each neuron. Note that *r*_0_ enters *μ*, but it does not appear in *σ* ^2^. *ι* is the time constant of firing rate dynamics. In these equations *k*_*i*_ is the corresponding coefficient of each *r*_*i*_ in Eq (2) and could be easily inferred form Eq (3) or Eq (4). *J*_*i*_ is a positive number reflecting the PSP amplitude of the presynaptic neuron *i*. In other words, in the EEI network, for example, *J*_1_ and *J*_2_ are either equal to *J* or *wJ*, depending on whether they reflect the PSP of neurons within or between population. *J*_3_ is equal to −*gJ* as it belongs to the inhibitory population.

We suggest an approximation for Eq (5) and we write the right-hand side in the form of a polynomial so that the analysis becomes simpler. We expect Eq (5) to be a function of rate (*r*_*j*_) of any presynaptic population. To see the dependence, we take the derivative of Eq (5) with respect to any *r*_*j*_. For that purpose, we use the chain rule for derivatives and we consider $\frac{\partial {\dot{r}}_{i}}{\partial r_{j}}=\frac{\partial {\dot{r}}_{i}}{\partial \mu }\frac{\partial \mu }{\partial r_{j}}+\frac{\partial {\dot{r}}_{i}}{\partial \sigma }\frac{\partial \sigma }{\partial r_{j}}$. Doing so, we will end up with

$$\begin{array}{ccc} \iota \frac{\partial {\dot{r}}_{i}}{\partial r_{j}} & = & \frac{-\sigma k_{j}\sqrt{\pi }-\left(\theta -\mu \right)\sqrt{\pi }\left|J_{j}k_{j}\right|/\left(2\sigma \right)}{\sigma^{2}\sqrt{\pi }}exp\left(-\frac{{\left(\theta -\mu \right)}^{2}}{\sigma^{2}}\right)\\ & & -2\frac{\theta -\mu }{\sigma }\frac{\theta -\mu }{\sigma \sqrt{(}\pi )}\frac{-k_{j}\sigma -\left(\theta -\mu \right)\left|J_{j}k_{j}\right|/\left(2\sigma \right)}{\sigma^{2}}exp\left(-\frac{{\left(\theta -\mu \right)}^{2}}{\sigma^{2}}\right) \end{array}$$ (6)

On the right hand side of the above equation, there are two terms that share a common factor $\frac{\theta -\mu }{\sigma \tau \sqrt{\pi }}exp(-\frac{{\left(\theta -\mu \right)}^{2}}{\sigma^{2}})$. From Eq (5), we know that this term is equal to the stationary rate *r*_*i*_. Therefore, we replace the common terms and get the following simpler equation

$$\begin{array}{c} \iota \frac{\partial {\dot{r}}_{i}}{\partial r_{j}}=r_{i}(\frac{-|J_{j}k_{j}|}{2\sigma^{2}}+\frac{2(\theta -\mu )}{\sigma^{2}}(k_{j}+\left(\theta -\mu \right)|J_{j}k_{j}|/\left(2\sigma^{2}\right))-\frac{k_{j}}{\theta -\mu }) \end{array}$$ (7)

Eq (7) indicates that the coupling from any presynaptic population to a given neuron in population *i* can be approximated in the following form

$$\begin{array}{c} \frac{\partial {\dot{r}}_{i}}{\partial r_{j}}=r_{i}k_{j}\left(c_{1}+\text{poly}\left(\sum_{k}r_{k}\right)\right)+c_{2} \end{array}$$ (8)

where poly(.) indicates a polynomial function of *r*_*k*_ up to higher orders which could be obtained by Taylor approximation. Note that Eq (8) gives an expression for the relative change of the rate dynamics ${\dot{r}}_{i}$ with respect to *r*_*j*_. To get an equation for ${\dot{r}}_{i}$, one needs to use a multivariate Taylor approximation to be able to recover ${\dot{r}}_{i}$ from *r*_*j*_s, for *j* ∈ {0,1,2,3}. After some simplifications, the general form of neural rate dynamics for any population *i*, is

$$\begin{array}{ccc} \frac{dr_{i}}{dt} & = & r_{i}\left(\sum_{j}k_{j}\left(r_{j}+C_{j}\right)\left(c_{1}+\text{poly}\left(\sum_{k}r_{k}\right)\right)\right)+C_{2}\\ & = & r_{i}\left(\sum_{j}k_{j}r_{j}+m_{1}+\text{poly}\left(\sum_{k}r_{k}\right)\right)+m_{2} \end{array}$$ (9)

where *C*_*j*_ is the operating point of *r*_*j*_ and *C*_2_ = *c*_2_(∑_*j*_ *C*_*j*_) is obtained from the expansion. *m*_1_ = *C*_1_(∑_*j*_ *C*_*j*_ *k*_*j*_) and *m*_2_ = *C*_2_ are constant terms (Also, note that the coefficients of *r*_*k*_ inside *poly*(.) are different now).

So far, we considered the dynamics of individual neurons in each population. However, note that *r*_*i*_ can be replaced by *R*_*i*_/*N*_*i*_, as outlined before. Therefore, Eq (9), with rescaled parameters according to corresponding population sizes, holds for the population dynamics as well. Eq (9) is of Lotka-Volterra type with an additional constant *m*_2_. Essentially, it describes the first temporal derivative of the rate variable $\dot{r}$ in the form of the rate variable *r* multiplied by a polynomial function of all other rate variables in each population. Given the connectivity matrix that relates the input to the population rates, shown in Eqs (3) and (4), we write the dynamics of competition in the following form

$$\begin{array}{c} \left\{ \begin{array}{c} {\dot{X}}_{1}\left(t\right)=kX_{1}\left(t\right)\left(wX_{1}\left(t\right)+X_{2}\left(t\right)+k_{1}Y\left(t\right)+k_{0}+\text{poly}\left(X_{1}\left(t\right),X_{2}\left(t\right),Y\left(t\right)\right)\right)+h_{0}\\ {\dot{X}}_{2}\left(t\right)=kX_{2}\left(t\right)\left(X_{1}\left(t\right)+wX_{2}\left(t\right)+k_{1}Y\left(t\right)+k_{0}+\text{poly}\left(X_{1}\left(t\right),X_{2}\left(t\right),Y\left(t\right)\right)\right)+h_{0}\\ \dot{Y}\left(t\right)=k^{\prime }Y\left(t\right)\left(X_{1}\left(t\right)+X_{2}\left(t\right)+k_{2}Y\left(t\right)+k_{0}+\text{poly}\left(X_{1}\left(t\right),X_{2}\left(t\right),Y\left(t\right)\right)\right)+h_{0} \end{array}\right. \end{array}$$ (10)

where *X*_1_(*t*) and *X*_2_(*t*) are the firing rates of the two competing subnetworks, and *Y*(*t*) is the firing rate of the subnetwork with neurons of the different type. *k*, *k*′, *k*_0_, *k*_1_, *k*_2_ and *h*_0_ are constant parameters (Note that *k*_0_ = *m*_1_ and *h*_0_ = *m*_2_ in Eq (9)). In the case where the two competing subnetworks are comprised of excitatory neurons, we have *k*_1_ = −*pg*. *k* is a factor influenced by the size of the network, the PSP amplitude, the time constant of the membrane potential, and the number of connections between excitatory neurons. This parameter is positive for the EEI network, and it will take a negative sign for the EII network. In the latter case, the sign of *k*_0_ and *k*_1_ flips (compare elements of *W*_*EEI*_ and *W*_*IIE*_ in Eqs (3) and (4), respectively). *k*_0_ results from effective couplings between subnetworks and the constant current input. *h*_0_ is a constant used to make sure that, when any of *X*_1,2_(*t*) is equal to zero, the external drive to the network kicks the system out of the fixed point and the activity of the network does not die out. This is moreover justified by the constant external drive to the network.

We hypothesize that on the population level, as far as the slow dynamics are considered, the expression poly(*X*_1_(*t*), *X*_2_(*t*), *Y*(*t*)) in the dynamics of *X*_1_ and *X*_2_ in Eq (10) can be neglected and the other terms, which are linear combinations of population firing rates (meaning *wX*_1_(*t*) + *X*_2_(*t*) + *k*_1_ *Y*(*t*) and *X*_1_(*t*) + *wX*_2_(*t*) + *k*_1_ *Y*(*t*)), can be replaced by *μ*_*i*_ in Eq (2) to get a qualitative behavior of the system. In other words, we examine the mean-field coupling weights between subnetworks as the coupling coefficients in the LV equation. This assumption simplifies the equations and it will be shown in the Results section that it does not distort the qualitative dynamics of the spiking network. As it turns out, however, due to small variability of the population with different neural type, poly(.) in Eq (9) cannot be neglected for the dynamics of $\dot{Y}$ (see the Results section). We will demonstrate later that plugging relevant parts of the coupling matrices *W*_*EEI*_ and *W*_*IIE*_ shown in Eqs (3) and (4) into Eq (10) indeed leads to a dynamical system that, on a population level, qualitatively behaves the same as the simulated large-scale network.

We define two new variables which are related to the activities of the competing subnetworks, *X*_1_(*t*) and *X*_2_(*t*).

$$\begin{array}{ccc} X_{1}\left(t\right)+X_{2}\left(t\right) & = & 2C(t)\\ X_{1}\left(t\right)-X_{2}\left(t\right) & = & 2D(t) \end{array}$$ (11)

where *C*(*t*) is the average firing rate of the two competing subnetworks and *D*(*t*) is half the difference between the two rates. With these new variables, we can rewrite Eq (10) by replacing *X*_1_(*t*) = *C*(*t*) + *D*(*t*) and *X*_2_(*t*) = *C*(*t*) − *D*(*t*)

$$\begin{array}{c} \left\{ \begin{array}{c} \dot{C}\left(t\right)=C\left(t\right)\left(k_{0}+k\left(\left(w+1\right)C\left(t\right)+k_{1}Y\left(t\right)\right)\right)+k\left(w-1\right)D\left(t\right)+h_{0}\\ \dot{D}\left(t\right)=D\left(t\right)\left(k_{0}+k\left(2wC\left(t\right)+k_{1}Y\left(t\right)\right)\right) \end{array}\right. \end{array}$$ (12)

It will be shown later in the Results section that *Y*(*t*) is well approximated by a polynomial function of *C*(*t*) and *D*(*t*). More specifically, data analysis of network simulations suggests the following relationship between *Y*(*t*) and the new variables *C*(*t*) and *D*(*t*)

$$\begin{array}{c} Y\left(t\right)=a+bC\left(t\right)+fD^{2}\left(t\right) \end{array}$$ (13)

As it is obvious from Eq (12), *D*(*t*) = 0 is a fixed point of the system. However, its stability depends on the parameters of the system. This means that the zero difference between the firing rates of the competing subnetworks (equal activity) is always a solution, however, it might be situated like a mountain pass (saddle node) between other stable solutions (valleys) of *D*(*t*), which will manifest itself in the observed switching dynamics of the population rates.

### Lyapunov function

In the previous section, we suggested a dynamical system that had two stable nodes which were separated by a saddle point. Such a system will relax to one of the stable nodes, when the external input is constant. These fixed points reflect the macroscopic dynamics of the network on the large scale. However, due to the finite number of neurons in the network and synaptic interactions between them, the stationary solution of the system is accompanied by marked fluctuations around the fixed point, which is the manifestation of microscopic dynamics in a high-dimensional system. This so-called “self-generated noise” or “intrinsic noise in the system” kicks the trajectory away from one fixed point and, passing by the saddle point, the trajectory will be lead to the other stable fixed point. This transition causes the switch from high to low activity (or *vice versa*) of a population. To gain a qualitative understanding how the average life time of the active state depends on the bifurcation parameter *w*, one way is to find the energy function of the system and check how the difference between the energy of the different equilibrium points changes with the parameter. Assuming additive noise with a constant intensity in the dynamics, the bigger this difference is, the less probable the occurrence of a state switching. As it will be demonstrated in the Results section, the influences of *h*_0_ in Eq (10) and *f* in Eq (13) on the dynamics of Eq (10) are extremely weak. Therefore, for mathematical simplicity, we neglect them in order to get a Lotka-Volterra set of equations in their generalized form.

We use a Lyapunov function as a specific implementation of such an energy function. It should be emphasized, though, that the Lyapunov function is not unique, and the interpretation as an “energy” function of the system should be taken as a metaphor, rather than as a rigorous physical statement. The system Eq (10) in two dimensions is not integrable and a strict-sense energy function does not exist. A Lyapunov function for *n*-dimensional competitive Lotka-Volterra equations has been suggested in [62]. Here, we use the same approach to obtain an estimate of an energy-like quantity in the (*X*_1_, *X*_2_) state space. According to [62], for the following general form of Lotka-Volterra equations

$$\begin{array}{c} \frac{dX_{i}}{dt}=p_{i}\left(X_{i}\right)\left(q_{i}\left(X_{i}\right)-\sum_{k=1}^{n}c_{ik}d_{k}\left(X_{k}\right)\right) \end{array}$$ (14)

one possible Lyapunov function is

$$\begin{array}{c} V\left(X\right)=-\sum_{i=1}^{n}\int_{0}^{X_{i}}q_{i}\left(z_{i}\right)d_{i}^{\prime }\left(z_{i}\right)dz_{i}+\frac{1}{2}\sum_{j,k=1}^{n}c_{jk}d_{j}\left(X_{j}\right)d_{k}\left(X_{k}\right) \end{array}$$ (15)

The simplified 2*D* equations for the dynamics of interaction between the two excitatory populations in the EEI case (wherein *Y*(*t*) is replaced by (see Eq (13)) *a* + *bC*(*t*) + *fD* ^2^(*t*), *f* ≈ 0) are the following

$$\begin{array}{c} \left\{ \begin{array}{c} {\dot{X}}_{1}\left(t\right)=kX_{1}\left(t\right)\left(wX_{1}\left(t\right)+X_{2}\left(t\right)-pg\left(a+\frac{b}{2}\left(X_{1}+X_{2}\right)\right)+k_{0}\right)\\ {\dot{X}}_{2}\left(t\right)=kX_{2}\left(t\right)\left(X_{1}\left(t\right)+wX_{2}\left(t\right)-pg\left(a+\frac{b}{2}\left(X_{1}+X_{2}\right)\right)+k_{0}\right) \end{array}\right. \end{array}$$ (16)

with a nontrivial fixed point at $\frac{pga-k_{0}}{w+1-pbg}(1,1)$ and two stable fixed points at $\left(0,\frac{pga-k_{0}}{w-0.5pgb}\right)$ and $\left(\frac{pga-k_{0}}{1-0.5pgb},0\right)$ on the *X*_1_ and *X*_2_ axis. It leads to the following Lyapunov equation

$$\begin{array}{c} V\left(X1,X2\right)=-\left(k_{0}-pga\right)\left(X_{1}+X_{2}\right)+\left(\frac{pbg}{2}-w\right)x_{1}^{2}+\left(\frac{pbg}{2}-w\right)X_{2}^{2}+X_{1}X_{2}\left(\frac{pbg}{2}-1\right) \end{array}$$ (17)

with a derivative function which obeys

$$\begin{array}{ccc} \dot{V}\left(X1,X2\right) & = & -kX_{1}{\left(wX_{1}\left(t\right)+X_{2}\left(t\right)-pg\left(a+\frac{b}{2}\left(X_{1}+X_{2}\right)\right)+k_{0}\right)}^{2}\\ & & -kX_{2}{\left(X_{1}\left(t\right)+wX_{2}\left(t\right)-pg\left(a+\frac{b}{2}\left(X_{1}+X_{2}\right)\right)+k_{0}\right)}^{2} \end{array}$$ (18)

and therefore, is negative semidefinite.

## Results

In this section, we present simulation results for the two case studies and compare them with the model based on Lotka-Volterra equations, as described in the Methods section. When we consider a typical system in the switching regime, the parameters for the scenario with two excitatory one inhibitory population (EEI) are *J* = 0.1 mV and *w* = 2.5 and the parameters for the scenario with one excitatory and two-inhibitory populations are *J* = 0.1 mV and *w* = 0.7. All networks under study are operated in the fluctuation driven regime, and the free mean membrane potential of neurons typically remains below threshold.

### Two-excitatory one-inhibitory network (EEI scenario)

In this case, two excitatory populations are competing with each other, and they are counterbalanced by one inhibitory population. As it will be shown later, for certain parameters of the system, switching dynamics between the two excitatory populations will occur. In this section, the nonlinearities governing the dynamics of the system, which are inferred from simulation results, as well as predictions from the corresponding Lotka-Volterra model will be demonstrated. In the case of switching behavior of the system, the life time distribution of the active states as a function of the bifurcation parameter *w*, as well as a Lyapunov function (energy landscape) of the deterministic dynamics will be described.

#### Switching dynamics as a collective emergent property

**Fig 2:**
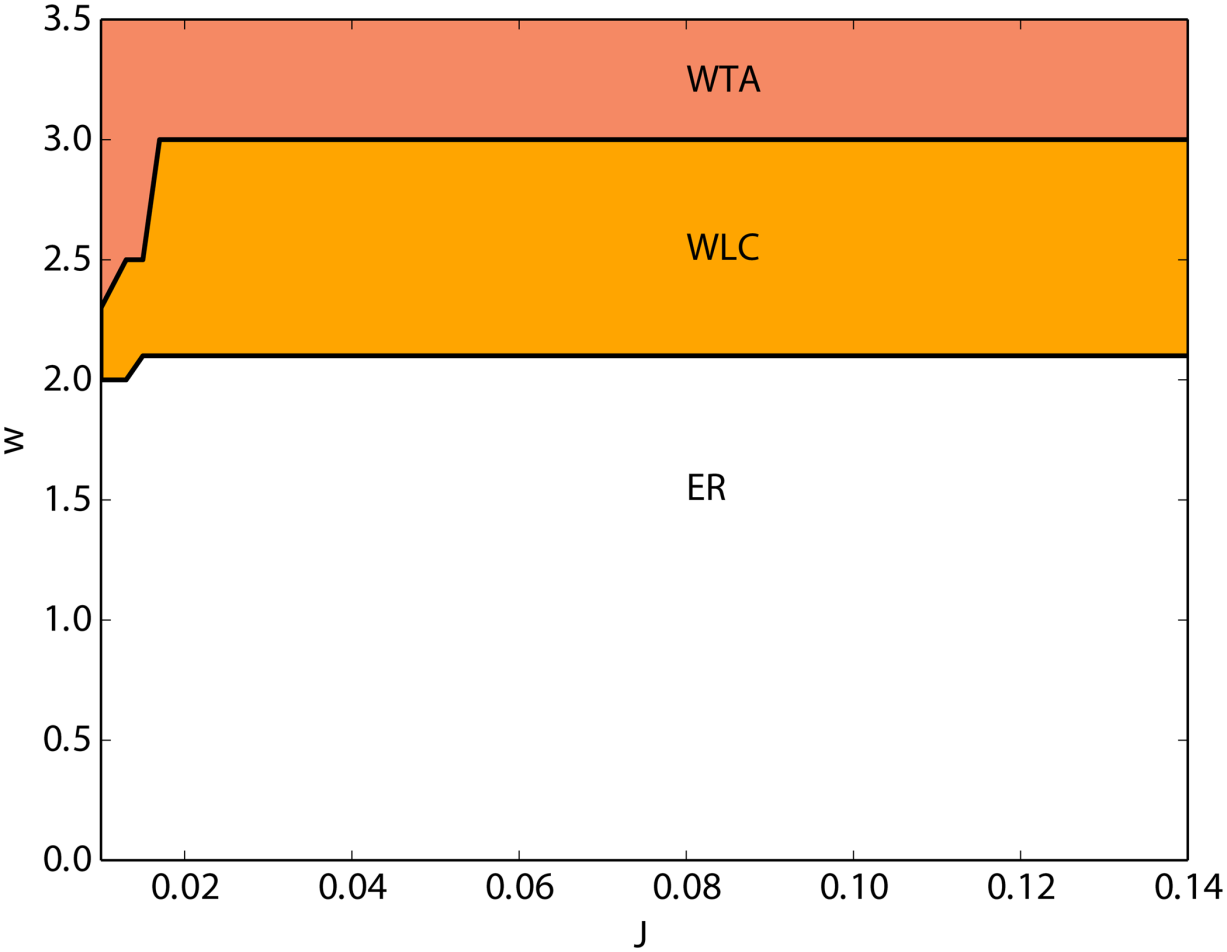
In the *J* − *w* parameter space, three different types of dynamical behavior is observed for the EEI scenario. (1) Equal rate (ER) for small values of *w*, in which the two excitatory subnetworks have identical non-zero firing rates. (2) For intermediate values of *w*, the two identical populations keep switching the activity between themselves, and none of them maintains the high activity, so called “winner-less competition” (WLC). (3) Large values of *w* result in winner-take-all (WTA) dynamics in which, depending on the initial conditions, one of the two competing populations maintains the high activity.

In our simulation studies, we considered the effects of *J*, the amplitude of excitatory to inhibitory postsynaptic potentials (PSP), and *w*, the ratio between the excitatory PSP amplitude within each population and the excitatory to inhibitory PSP amplitude, on the network dynamics. As shown in Fig 2, for intermediate values of *w* the network undergoes a switching dynamics between the two excitatory populations. As none of the two excitatory subnetworks really wins the competition, we call this a winner-less competition (WLC) state. For values of *w* below 2 the firing rates of the two excitatory populations are non-zero and identical, and in Fig 2 it is labeled as “equal rate” (ER). For values larger than 3, depending on the initial conditions of the two populations, one of the excitatory subnetworks stays at a relatively higher activity and the other one has a very low activity. We call this a winner-take-all (WTA) state, as the dominating population keeps its activity up for the entire range of simulation time. The global state of the network depends mostly on *w*, and to the degree to which this can be observed, *J* does not seem to affect the dynamics. Therefore, in our study, we consider *w* as an essential parameter that plays role in the collective behavior of the network (bifurcation parameter).

**Fig 3:**
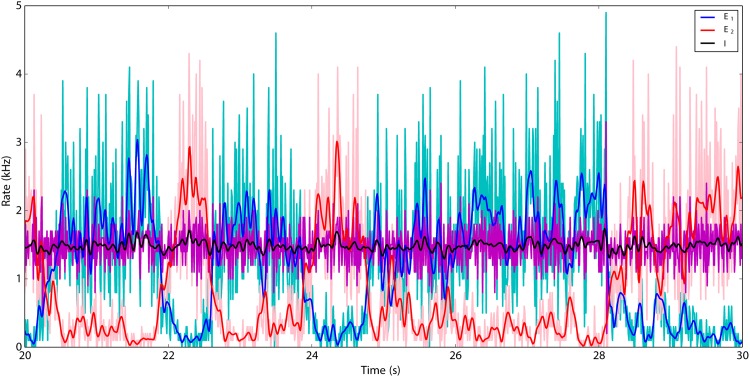
Switching dynamics of the two excitatory subnetworks for *J* = 0.1 mV and *w* = 2.5. The population histograms of the three subnetworks with time intervals of 10 ms are plotted in light colors. A Savitzky-Golay filter with length 2*n* + 1 = 21, *dt* = 10 ms and polynomial order *m* = 4 was used to smoothen the signals, which are plotted in darker colors. The activity of the inhibitory subnetwork has less fluctuations compared to the activity of the excitatory subnetworks.

To demonstrate the switching dynamics between the excitatory subnetworks, we chose *J* = 0.1 mV and *w* = 2.5 as the parameters of the network. The population histogram of the three population activities estimated from spike counts in time bins of 10 ms are shown in light colors in Fig 3. The low-pass filtered rates are displayed in darker colors. It is obvious from these data that the excitatory population rates are more variable than the inhibitory population rate. The switch takes place almost instantaneously, and the two excitatory populations never fire simultaneously at high rate.

#### Correlations and firing rate distributions

**Fig 4:**
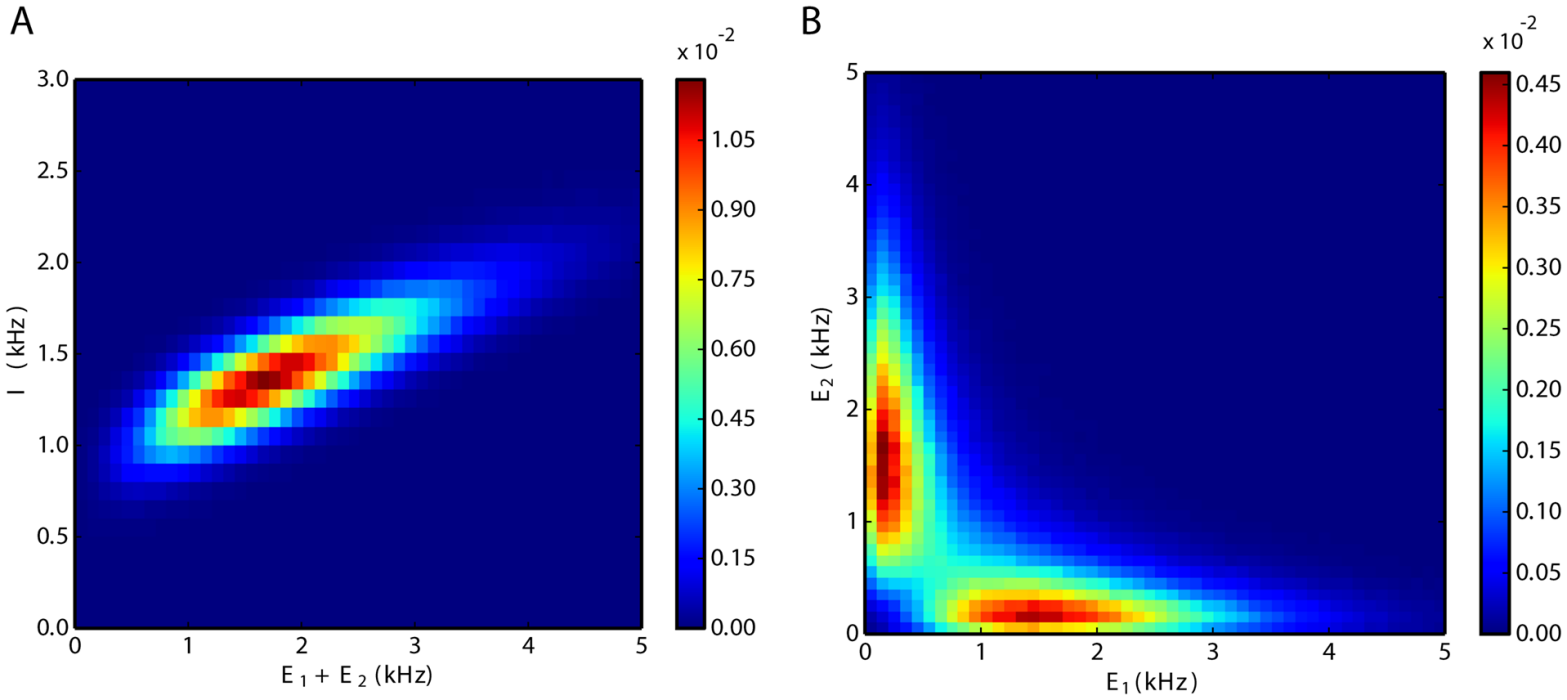
Joint distributions of population activities. **A**. Interactions between total excitation and inhibition. The elliptical shape of the distribution indicates a strong positive correlation between excitation and inhibition. **B**. Negative interactions between the two excitatory populations. As activity is almost mutually exclusive, the term switching seems adequate. The unit of the numbers on the color bars are ms^2^.

As far as the mean membrane potentials of individual neurons are below threshold and pairwise correlations are negligible, the overall network is in the balanced state. The balance is maintained during switching; therefore, the combined activity of the two excitatory populations is highly correlated with the activity of the inhibitory population (Fig 4A). For intermediate values of *w*, when switching dynamics emerges, the two excitatory populations are negatively correlated (Fig 4B). From the two-dimensional distributions of the activity of the excitatory populations it is clear that the activity of each excitatory population has a bimodal distribution.

Some studies on bistability of spiking networks with different neuronal dynamics rely on a bistable dynamics of individual neurons [63]. However, LIF neurons are not bistable elements, and for any fixed input statistics, there is a unique solution of the first-passage time problem [64, 65]. Therefore, the switching dynamics is not induced by single neuron dynamics, but it is a system property that emerges only in large-scale networks.

#### Nonlinear dynamics of interactions

**Fig 5:**
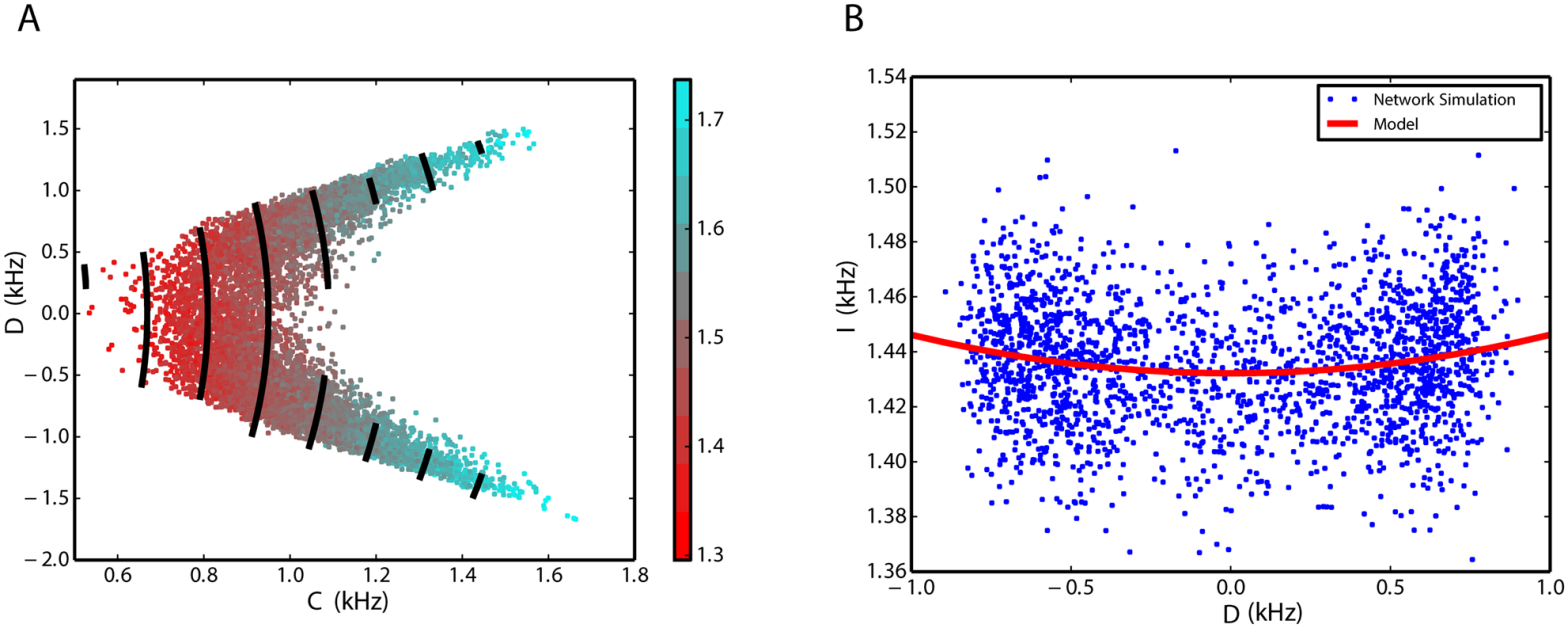
Inhibitory firing rate as a linear function of *C* and quadratic function of *D*. **A**. Data from numerical simulations of the network are illustrated as colored points. The color bar shows the firing rate of the inhibitory population in unites of spikes per milliseconds (same unites for *C* and *D*). Black contour lines show the isolines of Eq (13) after parameter estimation. The equal distance between the iso-lines indicates a linear relationship between the inhibitory firing rate and *C*. Unites of the numbers on the color bar are *kHz*. **B**. Data points for a constant level of *C* = 0.9 kHz are illustrated in blue dots as a scatter plot of *D* and *I*. Model fit for all available data points after plugging *C* = 0.9 into Eq (13), shows the quadratic dependence of *I* on *D* (red curve).

Simulation results show that the inhibitory rate is a function of the sum, *C*, and the difference, *D*, of the rates of the two excitatory populations (Fig 5). To make the relationship visible, we used a Savitzky-Golay filter (with parameters described in the Method section) to reduce the noise. Inhibitory firing rate scales linearly with *C* and quadratically with *D*. The linear relationship with *C* is clear from the equal distances between the black contour lines (model fit) in Fig 5A. Quadratic relationship with *D* is illustrated in Fig 5B for a constant value of *C*. Eqs (9), (10) and (13) imply that in general the rate dynamics can be approximated as polynomial functions of the firing rates of the involved subnetworks. Our simulation results show that for the inhibitory firing rate, the second-order terms of the excitatory rates play role in the dynamics, however, we can neglect them in the dynamics of the excitatory firing rates (see Eq (10)). For the specific values of *J* and *w* in this case study, we estimate *a* = 1.112 kHz, *b* = 0.356 and *f* = 0.014 ms in Eq (13), respectively, using general purpose least-square optimization available in the SciPy library [66]. A chi-square test for the goodness-of-fit results in a *p*-value of 10^−6^.

**Fig 6:**
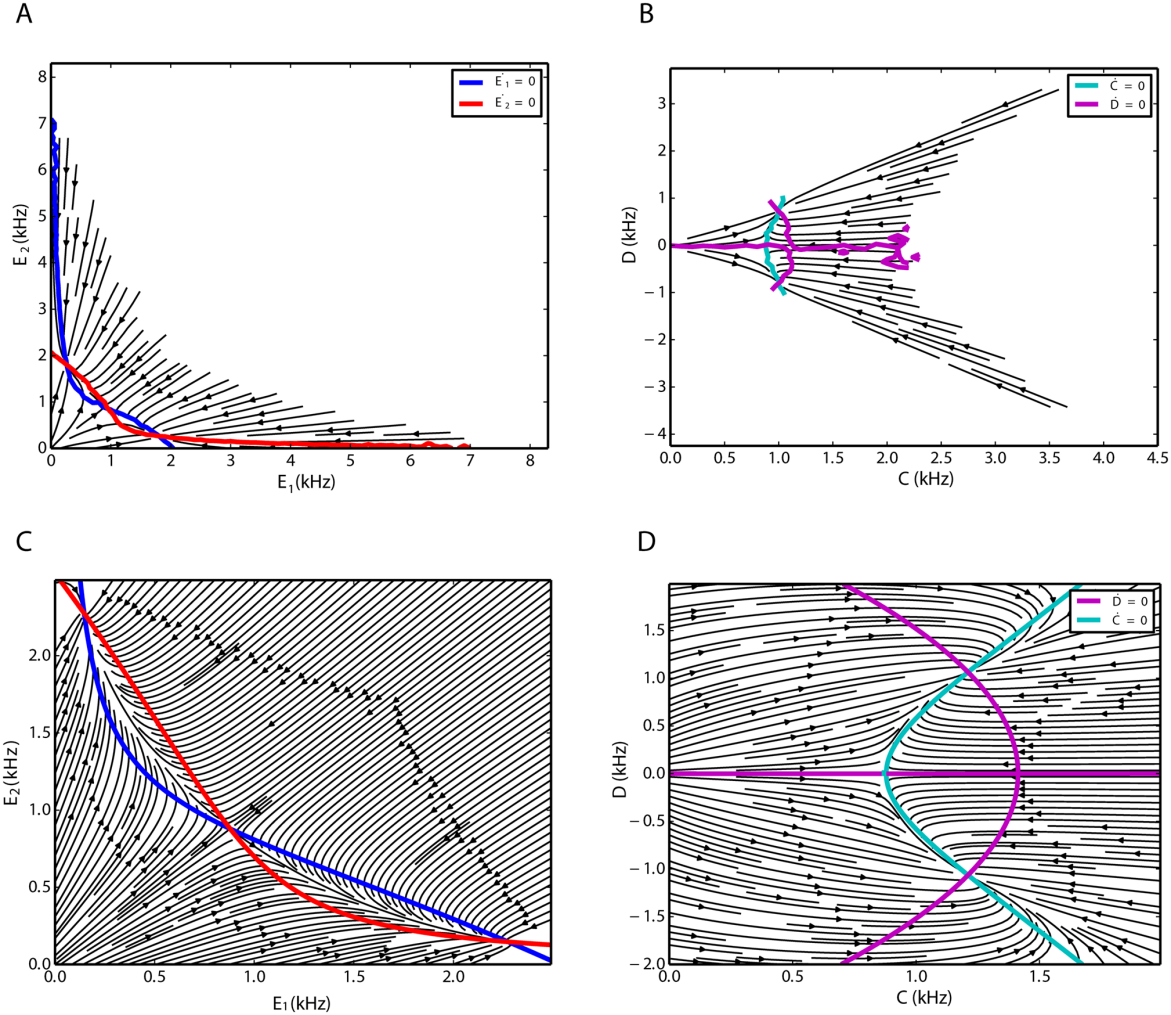
Flow of the two excitatory subnetworks in two dimensions, extracted from simulated time series (top) and inferred from the Lotka-Volterra model (bottom). **A**. State space of excitatory-excitatory firing rates extracted from simulations. **B**. Sum-difference state space extracted from simulations. **C**. State space of excitatory-excitatory firing rates are inferred from the model. **D**. Flow of the system in Sum-difference state space inferred from the model.

A population histogram with bin size of 10 ms was extracted for each of the three involved subnetworks. Using normalized first-order differences, we estimated the corresponding derivatives of all three signals. The fluctuations of the inhibitory population are negligible compared to the activities of the two excitatory populations. Moreover, we know the relationship between the omitted variable and the firing rates of the two excitatory populations, as demonstrated in Fig 5 (see also Eq (13)). Therefore, the projection to these two dimensions gives a good picture of the three-dimensional dynamics. In the two-dimensional reduced state space of the firing rates of the competing subnetworks, using the method used in [55], we estimated the deterministic flow of the two excitatory populations (Fig 6A). The nullclines of the system were obtained from the simulated time series by finding the zero-level contours of the derivatives in the two-dimensional state space. The corresponding flow of the network dynamics extracted form the time series in the transformed (*C*, *D*) state space is depicted in Fig 6B. The quadratic nature of the nullclines indicates that in Eq (10), second order polynomials are needed to represent the dynamics of the system properly.

The flows of the systems Eqs (10) and (12) are illustrated in Fig 6C, 6D, respectively. Using the parameters obtained from the mean-field analysis (section “Dimensionality reduction and inter-population couplings”), *k*_0_ = 22.0 kHz, *h*_0_ = 0.5 kHz, *k* = 1 ms, and *k*_1_ = −*pg*, the qualitative behavior of the model is very similar to the dynamics inferred from the network simulation (Fig 6A, 6B). In both cases, in the partial state space spanned by the firing rates of the two excitatory populations, two attractors are separated by a saddle node. In the (*C*, *D*) state space, *D* = 0 is one branch of the nullcline of the dynamics of the difference, but it does not form any stable fixed point of the system dynamics. However, two symmetric intersection points of the *C* and *D* nullclines define two stable critical points of the system. Due to the symmetry of the system, the difference is a constant number but it has a different sign in each case. Different signs of *D* correspond to different temporary winners.

#### Special case of *w* = 1

**Fig 7:**
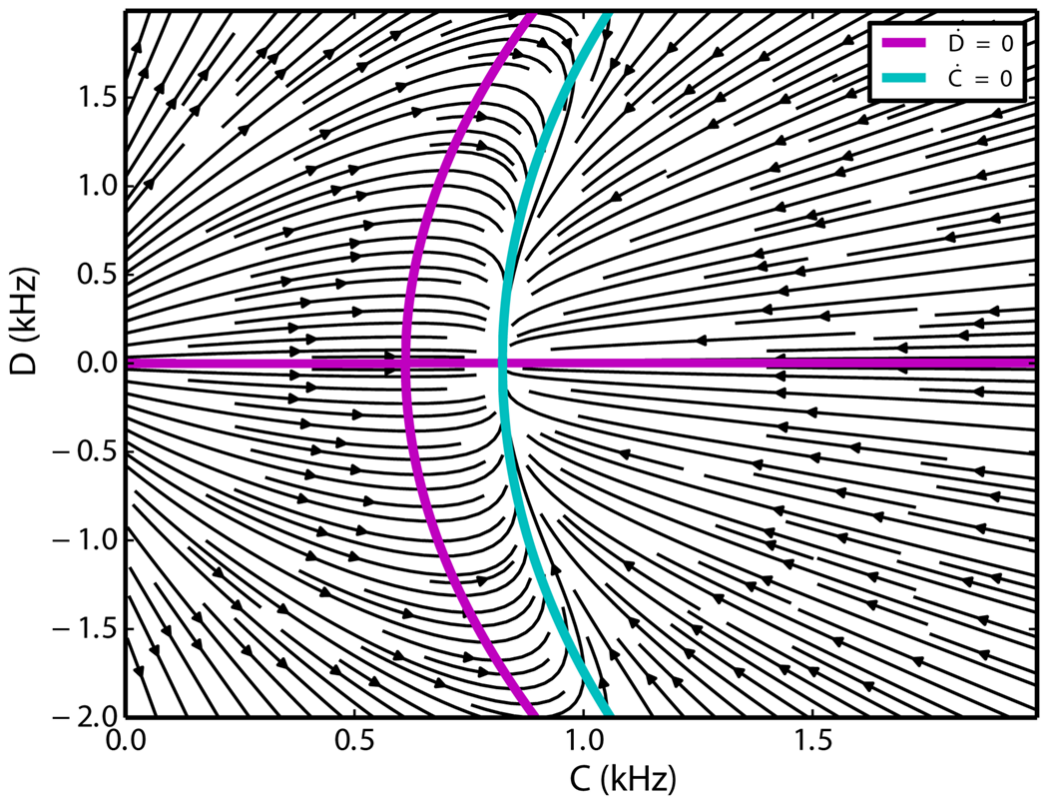
Flow of the system Eq (12) for *w* = 1. Only one fixed point at *D* = 0 exists, and the sum of the activities of the excitatory populations is nonzero. The equilibrium is unique, therefore stationary activity without any switching results.

In the special case of *w* = 1, there is no distinction between within and across connections of the excitatory populations and only one homogeneous excitatory subnetwork exists. In this case, the parameters of Eq (13) are *a* = 1.12 kHz, *b* = 0.27 and *f* = −0.01 ms, and the nonzero nullclines of *C* and *D* are parallel to each other. The *C* nullcline intersects with the *D* nullcline only in one point where it has to have *D* = 0 as a solution, as mentioned before, and *C* will have a nonzero value (Fig 7). This is the typical scenario in balanced random networks of excitatory and inhibitory populations, and the Lotka-Volterra model replicates this behavior for the parameters considered here. In this case, there is no difference between the firing rates of the two excitatory subnetworks and the system remains stationary (apart from random fluctuations). Also, in the low frequency regime, the inhibitory firing rate scales linearly with the excitatory activity (*f* is very small). This behavior has been demonstrated before in many studies [67–69]. For this particular value of *w*, only one fixed-point exists and shows that *w* is indeed a bifurcation parameter, as it changes the qualitative behavior of the system.

#### How do the fixed points depend on *w*?

**Fig 8:**
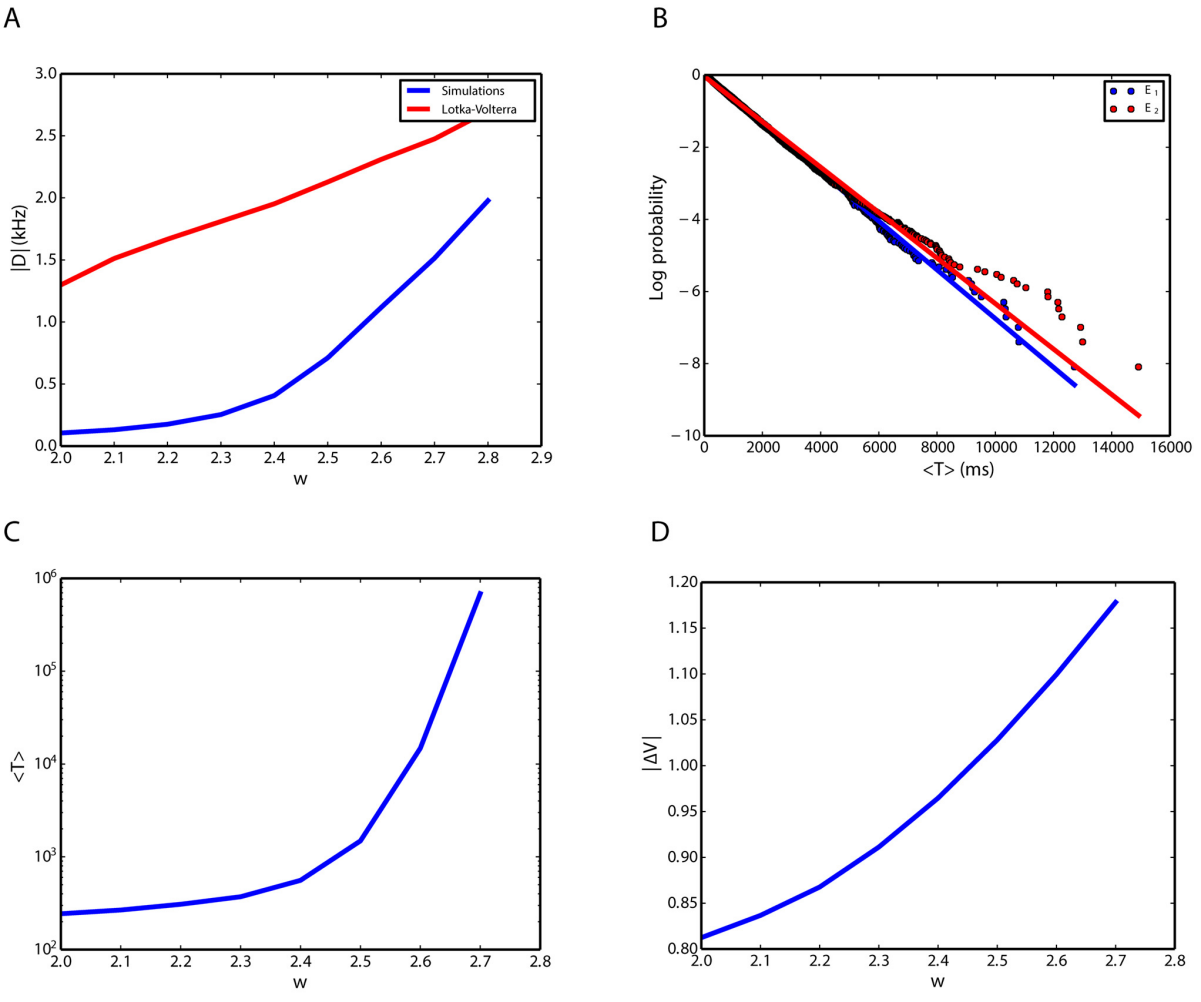
Life time statistics of the winning (high rate) population. **A**. Difference between the rates at the stable fixed point as a function of the parameter *w* that describes the relative strength of synapses within each population. **B**. Survivor function of the distribution of life times for the two competing excitatory populations. **C**. Average life time as a function of *w* for network simulations. **D**. Difference of energy levels (see text for details) between the attractor and the saddle point of Eq (10), when the inhibitory population firing rate is replaced by the other two dynamical variables, as a function of *w*.

Network simulations suggest that the bigger *w* is, the bigger the difference between the firing rates of the two competing populations is (Fig 8A). This means that if the neurons within each subnetwork interact with stronger synapses, the difference between the collective rates of the two competing populations becomes more pronounced. This shows that cooperation within populations makes a higher contrast between the two activity levels. In network simulations, the difference between the activity of the populations becomes obvious only for values above *w* = 2. For 1 < *w* < 2, there is no clear separation between the firing rates of the two excitatory populations. In fact, considering the size of the network, “within” and “between” population weights are not distinct enough to manifest their effect on the collective dynamics in the network simulations. The Lotka Volterra model Eq (10) exhibits distinct stable fixed points for the entire range of positive *w* > 1 in our study. In Eq (10), the two excitatory populations are actually cooperating because they both have a positive influence on the growth rate of the other population’s activity. However, the farther apart the positive coefficients corresponding to each population activity are, the farther apart the two fixed points become.

#### Distribution of life times

As mentioned before, spontaneous switching in an attractor network does not take place unless there is some source of noise in the system. Here, we first show that the statistics of the switching times suggest that they are completely random. Then, we study the qualitative influence of parameter *w* on the life time statistics. This helps us to better understand the network behavior, represented in Fig 2. Moreover, we show that the noise dynamics is not additive, but strongly state dependent.

The survival distribution of the life times of the active state (times between two consecutive switches) could be very well approximated by an exponential function. This is illustrated in Fig 8B by fitting a straight line to the logarithm of the survivor (log-survivor) function of the dwell times that were inferred from network simulations (The survivor function for a random life time is a function that gives the probability that the stochastic system will survive beyond the time specified). This implies an exponential dwell time distribution. This provides a strong hint that the switches take place randomly (i.e. following Poisson statistics), and that the intrinsic noise (fluctuations due to the complex microscopic dynamics) in the network plays an important role for the statistics of the dwell times.

As a function of *w*, the average time between switches grows faster than exponential (Fig 8C). To illustrate the relationship between average life times and the coupling parameter *w*, we used the Lyapunov function of the system Eq (10) with *h*_0_ = 0 and *f* = 0 in Eq (13), as explained in the Methods section. The system was reduced to a two dimensional model by replacing *Y*, the inhibitory rate, in Eq (10) with the expression in Eq (13). Then, we calculated the energy difference between the attractor point and the saddle point. Intuitively, the bigger this difference, the more difficult it is for fluctuating forces to drive the system out of the attractor valley “uphill” towards the saddle. This difference as a function of *w* is plotted in Fig 8D and supports the idea of average life times growing with *w*. As a consequence, a winner-take-all behavior of the network emerges if *w* is large enough (Fig 2). In this regime, the energy difference between the stable node and the saddle point is large and the fluctuations of the population activity are not sufficient to enter the basin of attraction of the other stable fixed point.

**Fig 9:**
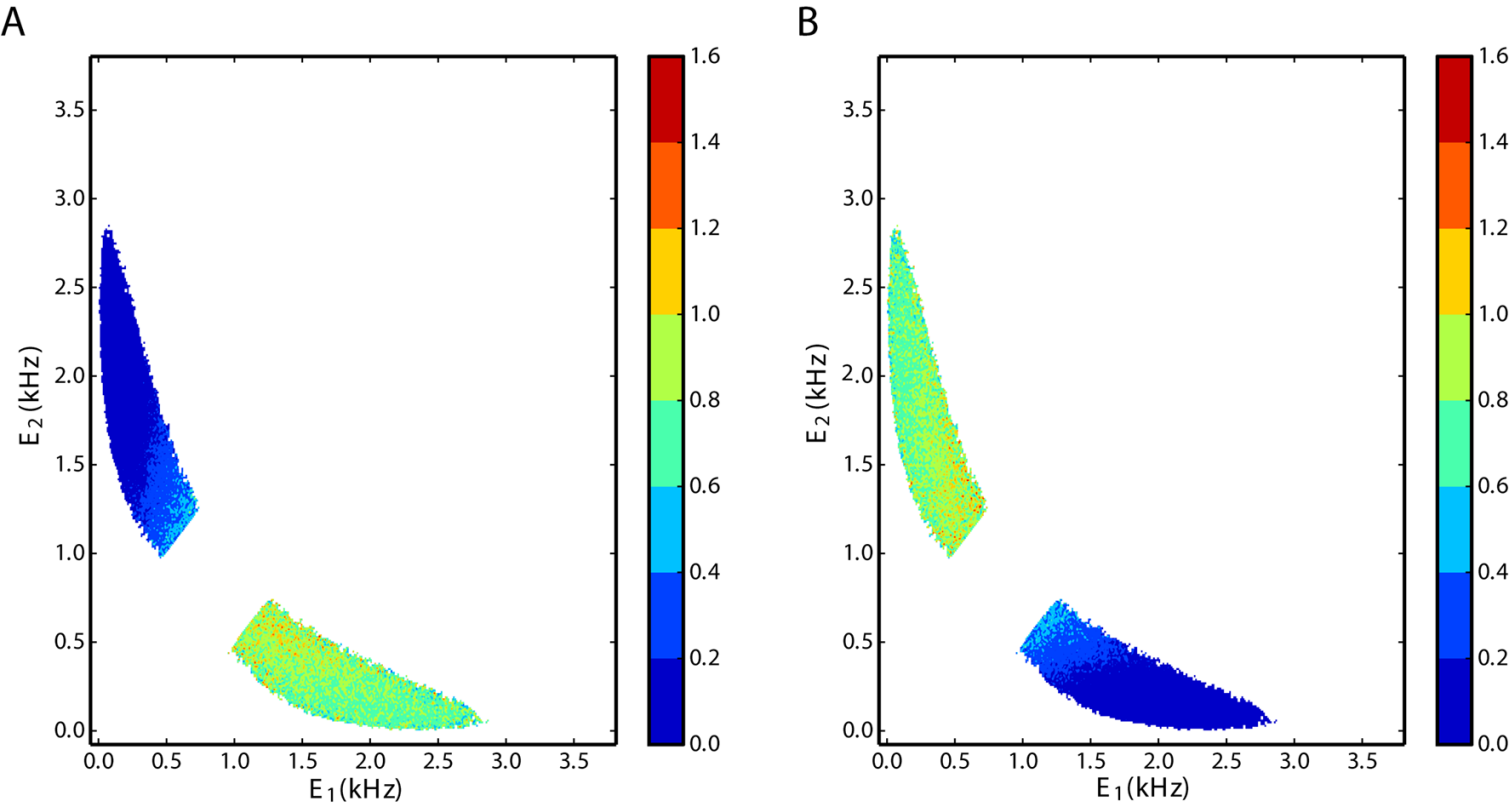
Variance of the noise in the state space of the two competing excitatory subnetworks. **A**. Variance of ${\dot{E}}_{1}$ and **B**. Variance of ${\dot{E}}_{2}$ in the state space spanned by *E*_1_ and *E*_2_.

As it is obvious, the attractor model suggested by deterministic Lotka-Volterra equations cannot represent switching dynamics but the existence of some intrinsic noise or random external perturbation is required. We stress that in the case considered here, the noise does not come from external sources. It reflects the high dimensional complex dynamics which cannot otherwise be represented by the low-dimensional description of the three population Lotka-Volterra model. It has been shown that this type of intrinsic noise arising in balanced random networks is multiplicative [55], and the fluctuations of the activity are proportional to the rate. In other words, random excursions into the direction of the active state are more likely to cause the alternation of the activity. Using the same method that was developed previously [55], we estimated the variance of the noise in the state space spanned by *E*_1_ and *E*_2_, which are the low-pass filtered firing rates of the two excitatory populations (Fig 9). At the moment that a switch from the high to the low rate takes place, the local variance of the signal increases drastically. To avoid the influence of switches on the variance in the state space, we did not display the respective data points around the saddle point of the dynamics (see saddle point in Fig 6A). The criterion for not showing the data points was based on the absolute difference between the firing rates of the two excitatory populations being inferior to some value, that was defined as a threshold. We found that if the data points that belong to a threshold of 0.5 spike/ms and less are not displayed, switching points will not influence the state space representation of the variance. In Fig 9A, the variance of the noise for the first excitatory subnetwork, *E*_1_, is shown. The larger the transient rate of *E*_1_, the larger the variance of the noise is. If the noise was additive, the variance in the state space would not change with the state of the firing rates of the populations and it would be unique for any state. Since the variance is not uniform in the state space, it is state dependent, and since it increases with the corresponding dynamical variable, it suggests a multiplicative noise model. Fig 9B shows the variance of the noise for *E*_2_ and the same behavior is observed for this variable.

In summary, the deterministic dynamics of the network, controlled by parameter *w*, also affects the stochastic behavior of the collective network dynamics. As our results show, increasing *w* reduces the chance of hitting the basin of attraction of the other stable fixed point and, as a consequence, the mean life time increases. This, justifies the idea that the intrinsic self-generated fluctuations in the network (microscopic dynamics), together with the stable fixed points (macroscopic dynamics) can explain the observed switching dynamics in the network simulations.

### Two-inhibitory one-excitatory network (EII scenario)

Most results for this type of network are similar to the EEI scenario. For values of *w* smaller than 1, the switching dynamics begins to manifest itself. As *w* decreases more, the mean of the life times become larger. The reason that the switching dynamics shows up for values of *w* between 0 and 1 is that the inhibitory subnetworks inhibits the neurons inside their own population less than any other neuron in the competing subnetwork. The exponential distribution of the life times for any value of *w* in this interval is still valid and the first moment of the life time decrease with increasing *w* (data are not shown). However, there is an interesting change in the shape of the nullclines of the two dynamical variables, which will be studied in the following section.

#### Nonlinear dynamics of interactions

**Fig 10:**
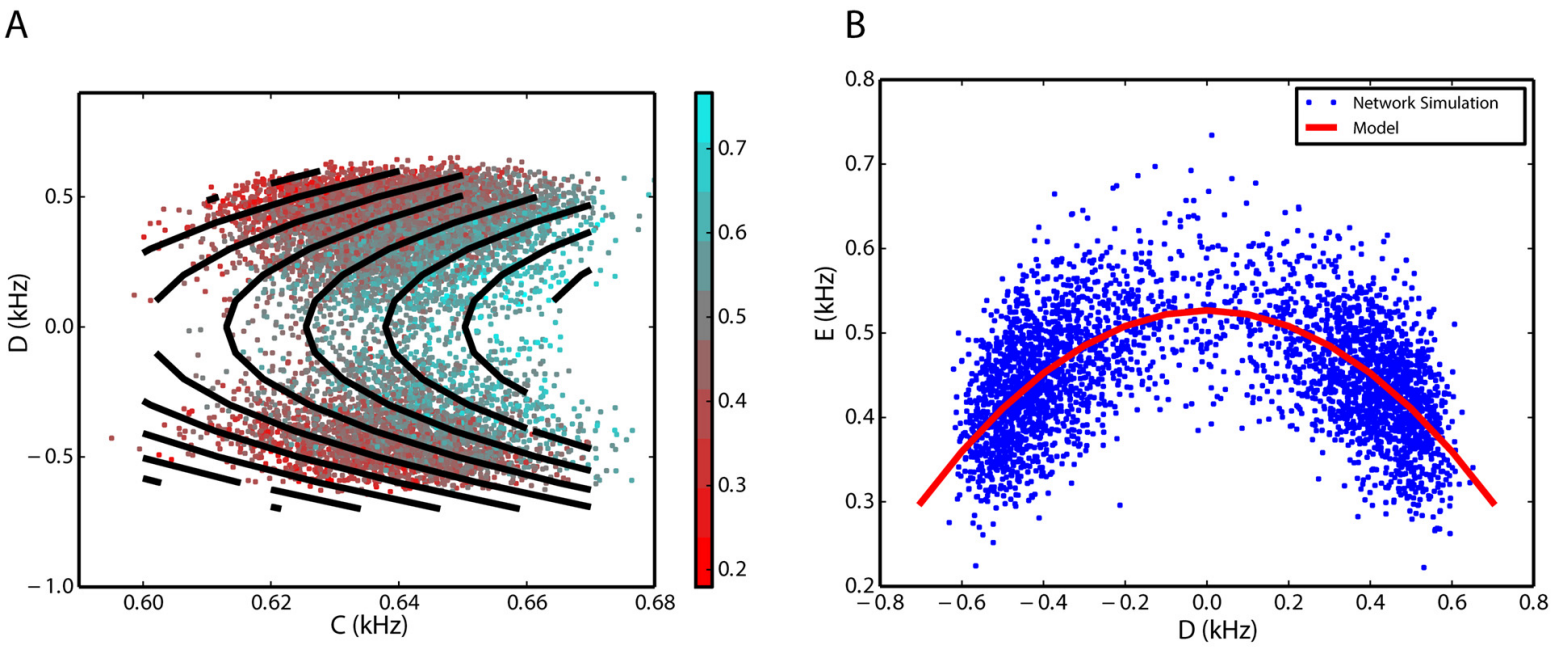
Excitatory population firing rate in the *C* − *D* coordinates, inferred from simulation results for a network in the EII scenario. **A**. Excitatory firing rates corresponding to different values of *C* and *D* are shown in different colors (see the color bar). Parameter estimation for Eq (13) shows the estimated values of the excitatory firing rate. The isolines for the model are depicted in black. **B**. Excitatory firing rate for a constant value of *C* = 0.64 kHz are illustrated in blue dots. For this constant, Eq (13) predicts a quadratic scaling of the excitatory rate with the dynamical variable *D*. The red curve is the model prediction, with parameters mentioned in the text, for the constant value of *C*. The dependence of the excitatory firing rate on *C* and ∣*D*∣ are positive and negative, respectively.

The two variables *X*_1_ and *X*_2_ in Eq (10), are now the dynamical variables for the two competing inhibitory populations, and *Y* is the variable for the firing rate of the excitatory population. Fig 10 illustrates the three dimensional scatter plot of the reduced system, where the third dimension (the firing rate of the excitatory population) is color-coded. Similar to Fig 5, the quadratic dependence of the excitatory firing rate on *D* and its linear dependence on *C* is appreciable. However, in this case study, the activity of the excitatory subnetwork decreases with increasing *D*. The parameters of equation *Y* can be estimated by least-squares regression method (*a* = −1.532 kHz, *b* = 3.217 and *f* = −0.464 ms with *p*-value = 10^−6^).

**Fig 11:**
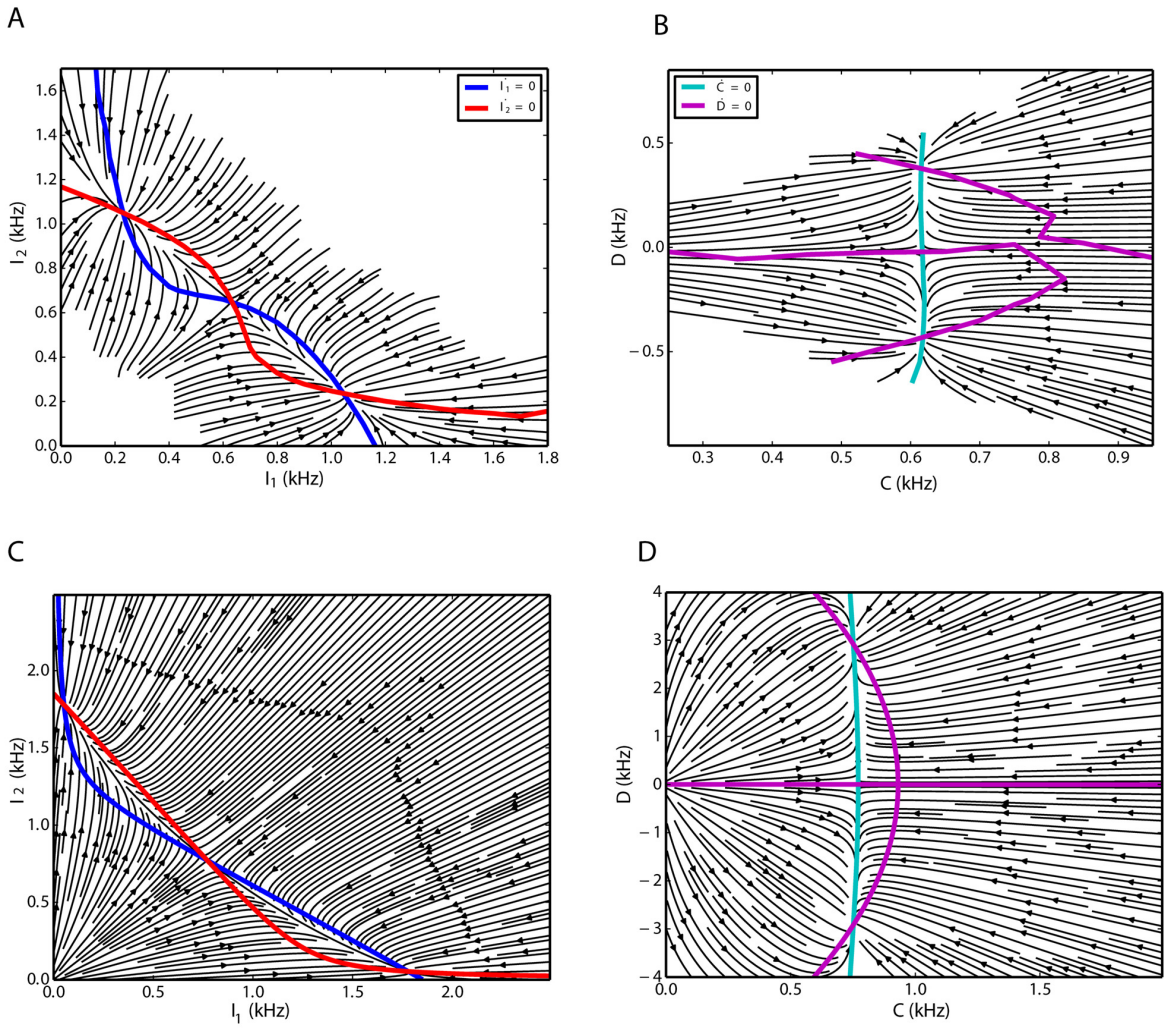
Two-dimensional flow characterizing the firing rate dynamics for an EII network. **A,C**. Excitatory-excitatory firing rate state space. **B,D**. Sum-difference state space.

Simulation results show that the network with two competing inhibitory subnetworks and a shared excitatory subnetwork also has two stable fixed points that are linked together via the repelling manifold of a saddle node in between (Fig 11A). The analogous behavior was described for the EEI scenario (Fig 11C). The flow in the state space spanned by the two inhibitory populations, inferred from the time series of a network simulation, indicates that the *C* nullcline does not depend on *D* (Fig 11B). This is exactly the same behavior as the one described by the Lotka-Volterra set of equations (Fig 11D).

## Discussion

We aimed at analyzing the competition dynamics between excitatory and inhibitory subnetworks embedded in a network in the balanced state. Such analysis is particularly useful for understanding the global dynamics of interactions between different brain regions, or between cortical columns in a small region. Starting with the mean-field approximation derived from a diffusion approximation [61, 70], using polynomial approximations, we derived a variant of the well-known generalized Lotka-Volterra equations. These equations are powerful enough to be considered as canonical models [45]. We studied two different cases where excitatory and inhibitory populations were interacting. We mainly studied the effect of the within population connectivity weights. In both cases, switching dynamics was observed both in large scale spiking network simulations and in firing rate equations (attractor dynamics with noise). There was a close qualitative match between the nullclines of the dynamics reconstructed from network simulations and those corresponding to the rate equations, with the correct type of equilibrium points. Interestingly, Lotka-Volterra equations with two types of prey and one predator are known to exhibit switching dynamics for certain parameter configurations [71]; however, in predator-prey dynamics, the influence of the prey population on its own growth rate is negative (due to limited resources). Therefore, a comparison with models of general population dynamics has to be done with care.

The proposed rate model assumes one dynamical variable for each population. The two scenarios that we studied in this paper, were networks of three interacting populations. In general, analyzing a three dimensional system using two dynamical variables implies constraints which may give a distorted picture of the underlying dynamics. However, we observed that the variability of the population with the opposite identity is smaller compared to that of the competing populations. The dynamics of the former population could be modeled as a second order polynomial function of the other two population activities. The same kind of dimensionality reduction has been applied in similar studies [10, 12]; however, the authors considered the stationary constant solution of the inhibitory firing rate without any fluctuations. In our first case study, we observed that inhibitory activity scales positively with the sum and the difference of the two excitatory populations. In the second case, however, excitatory firing rate scales positively with the sum and negatively with the difference of the two inhibitory firing rates. Because the overall system is in a balanced state, it is expected that the third non-paired population with a different neuron type must be positively correlated with the total activity of the other two populations [55, 67, 69]. Quadratic dependence, however, is a nonlinear effect and depends on the identity of the populations. Interestingly, in a similar study of binocular rivalry [19], the authors derived an energy function to describe the system dynamics to match experimental results, and they indeed ended up with a quadratic dependency of the inhibitory firing rate on the excitatory firing rates.

We emphasize that the switching dynamics observed in this study is a collective effect in a large-scale system, and it is not due to bistability of single neurons. In fact, LIF neurons with current based synapses have a unique response function for a given input. In other words, a stationary input, results in a stationary output firing rate. In [63], LIF neurons with conductance based synapses were the elements of a network with two excitatory and two inhibitory subpopulations. The stationary input-output function of a single neuron was shown to have a bistable property and as a result the whole network displayed switching dynamics. However, this justification does not apply to the networks that we considered in this paper.

We considered random networks with fixed in-degree and fixed out-degree (configuration model without any multiple or self connections) for the connectivity matrix of the network within and between populations in order to make sure that there are no hubs in the network and the switching dynamics is not induced by heterogeneities in the structure of the network. A previous study has shown that modifications to network topology (changed motif statistics), particularly in the inhibitory population, can result in bistable network activity [72]. In the network that we considered, all neurons had exactly the same number of inputs from each of the populations. Therefore, we can conclude that the switching dynamics is not induced by heterogeneities in degree distributions, but it is rather a collective network effect.

To come up with a collective rate equation, a dimensionality reduction is necessary. We chose the population histogram of each population activity as the signal representing the large-scale behavior of each component of the system. The histogram of the population activities of each network were derived from numerical simulations of these networks. From the simulated time series of the two competing subnetworks, we derived the mean state-dependent derivatives. From these data, we could extract the flow and the nullclines of the associated two-dimensional dynamics. We identified an equivalent Lotka-Volterra dynamics in two dimensions, which showed the same qualitative behavior in both case studies. The nullclines typically have three intersection points where two attractors are separated by a saddle node. The unstable manifold of the saddle redirects the trajectories towards the attractors. In a related study, it has been reported that when threshold-linear rate dynamics are considered for individual neurons, under some specific weight configurations, it results in the emergence of permitted and forbidden states in a random network [73–75]. In these networks, differential modes (eigenvectors with components of different sign) of the system are unstable, but the common modes (eigenvectors with elements of the same sign) are stable. The network as a whole is stable; however, multistabilities arise due to unstable differential modes. In our model, obtained from a reduction of a high-dimensional system, we can think of each competing population as a single set. The differential mode is on the unstable manifold of the saddle and the common mode is located on the stable manifold. Therefore, with a similar argument as [74, 75], we conclude that for some values of the bifurcation parameter, two populations of the same type cannot have high firing rates at the same time because it is not “permitted”, in the sense that it is not a stable solution. Another interesting conclusion of [73] is that a digital circuit made of CMOS transistors with shared inhibition among excitatory components is characterized by Lotka-Volterra equations that are able to capture a number of different phenomena in the cortex. In both cases (the circuit and the equations), the multistability due to the nonlinearity of its elements and the hybrid nature of the system result in a rich dynamics. Flip-flop behavior in the brain [76, 77] and computation by switching [78] are interesting ideas that have been suggested recently and Lotka-Volterra equations seem to be able to implement those kind of dynamics as well [73].

The attractor dynamics suggested in this paper, needs a source of fluctuations in order to be able to capture the switching dynamics. In the analysis of the data from network simulation, we observed that the life time distributions of the active states are well approximated by an exponential distribution. A similar observation was reported in [63], where LIF neurons with conductance based synapses in a different network configuration were studied. Our interpretation of this is that the “intrinsic noise” in the system, which mainly comes from finite size of the system and from the correlations between neurons, drives the system. The external source of input has no role here, as it is a constant current with no fluctuations. Finite-size fluctuations as a source of perturbation from stable fixed points of the network dynamics has, in fact, been suggested before [19, 54]. In our study, however, we had a closer look at the nature of the noise and we found that it is multiplicative rather than additive (see [55] for more detailed study). This feature makes the analysis of the relationship between the mean life time and the parameter *w* very difficult. However, intuitively and analytically, when the factor *w* increases in the EEI network, the distance between the fixed points increases, and the energy difference between the stable node and the saddle in the Liapunov function increases. This means that jumping over the barrier to reach to the basin of attraction of the other attractor becomes more difficult. A similar phenomenon occurs in the EII network. However, in this case the smaller *w* is, the longer the mean life time of the active state becomes. The reason is with smaller *w*, inhibitory neurons inside each cluster inhibit each other less as compared to the inhibition that they impose on the competing population. As a consequence, they cooperate more with each other. We remind the reader that there is no unique Lyapunov function of the system, but as the two-dimensional system was not integrable, we used the energy function suggested by [62].

We propose that Lotka-Volterra equations could be an alternative model for Wilson-Cowan equations in the low firing rate regime of balanced random networks. Both are capable of describing different types of dynamic features, including attractors, limit cycles and chaotic dynamics. Phenomena related to cortical dynamics, like decision making, binocular rivalry and persistent activity in working memory, could as well be justified by an attractor model, and in this paper we demonstrated an application of Lotka-Volterra equations for such modeling. Oscillations in the brain as a result of interaction between excitation and inhibition as well as central pattern generators might also be captured by a limit cycle behavior of such equations, however, further investigation is required. Furthermore, such equations were suggested to capture the robust transient dynamics in the brain which are reliable for information coding [79]. It is still not clear, however, how many populations of interacting spiking neurons with which parameters are needed in order to be able to implement a winnerless competition through transient dynamics.

In this paper, we considered the scenarios of three subnetworks wherein at least one of the subnetworks was comprised of inhibitory neurons. We speculate that for a larger number of subnetworks with at least one inhibitory cluster, there should be a region in parameter space where switching dynamics between populations is observed. The role of inhibition in generating winner-take-all dynamics is very well-known [46, 80]. In the large-scale dynamics of the network, inhibition makes an effective negative coupling from one excitatory population to the other one [19, 41]. Switching dynamics in the purely inhibitory networks of the striatum would be also a very interesting case to study. Investigating whether competitive Lotka Volterra equations can capture those dynamics is a topic of research to be considered. Moreover, in this paper, we considered a symmetric network in terms of connectivities. It would be interesting to see what happens for asymmetric networks and different coupling scenarios.
